# Hidden Markov Models based intelligent health assessment and fault diagnosis of rolling element bearings

**DOI:** 10.1371/journal.pone.0297513

**Published:** 2024-02-07

**Authors:** Yao Qifeng, Cheng Longsheng, Muhammad Tariq Naeem

**Affiliations:** 1 School of Economics and Management, Nanjing University of Science and Technology, Nanjing, China; 2 Department of Neurosurgery, Nishtar Medical College and Hospital, Multan, Pakistan; Wroclaw University of Science and Technology: Politechnika Wroclawska, POLAND

## Abstract

Hidden Markov Models (HMMs) have become an immensely popular tool for health assessment and fault diagnosis of rolling element bearings. The advantages of an HMM include its simplicity, robustness, and interpretability, while the generalization capability of the model still needs to be enhanced. The Dempster-Shafer theory of evidence can be used to conduct a comprehensive evaluation, and Stacking provides a novel training strategy. Therefore, the HMM-based fusion method and ensemble learning method are proposed to increase the credibility of quantitative analysis and optimize classifiers respectively. Firstly, vibration signals captured from bearings are decomposed into intrinsic mode functions (IMFs) using ensemble empirical mode decomposition (EEMD), and then the Hilbert envelope spectra of main components are obtained; Secondly, multi-domain features are extracted as model input from preprocessed signals; Finally, HMM-based intelligent health assessment framework and fault diagnosis framework are established. In this work, the life cycle health assessment modeling is performed using a few training samples, the bearing degradation state is quantitatively evaluated, normal and abnormal samples are effectively distinguished, and the accuracy of fault diagnosis is significantly improved.

## 1. Introduction

Rolling element bearings which consist of an inner ring, outer ring, cage, and rolling elements are important standardized components in rotary machines. Their typical applications include airplanes, automobiles, machine tools, and industrial robots. Bearing steel is one of the most high-standard alloy steels, and its production quality can be improved through process parameter optimization [1–3]. In industrial scenarios, heavy loads and high-intensity operating conditions make bearings highly susceptible to varying degrees and unpredictable damage such as cracks and pitting corrosion. The degradation of bearing performance may have adverse effects on the safe and stable operation of mechanical systems, leading to consequences such as shutdowns, accidents, and economic losses. Therefore, it is necessary to carry out bearing condition monitoring and condition-based maintenance (CBM). Due to the practical significance of rolling element-bearing health assessment and fault diagnosis technology for enterprise production, their theoretical and applied research have received much attention [4].

Vibration signal analysis is the foundation of data-driven rolling element-bearing health assessment and fault diagnosis, and feature extraction from the monitoring signals is a key procedure to further improve the model performance [5]. The vibration of bearings can be caused by external vibration sources, as well as by structural characteristics and defects. Usually, when a defect on one surface of a bearing strikes another surface, it triggers resonance in the bearing system, which is equivalent to modulating the fault signal. Due to the constraints of working speed and bearing geometry, the rotation of the inner or outer ring generates a series of continuous pulses. The signals exhibit nonlinear and non-stationary characteristics because of the system damping coefficient attenuation in the vibration transmission path, a clearance between the bearing outer race and the housing, sliding of a rolling element, and variable speed and load conditions [6]. In addition, measurement errors of the sensors installed on the bearing housing can only be reduced but cannot be eliminated, and background noise may come from other coupled machine components and the working environment [7]. These unfavorable factors have brought certain difficulties to signal analysis and processing.

To address this issue, some advanced methods have been proposed, such as variational mode decomposition (VMD) [8], wavelet packet transforms (WPT) [9], adaptive chirp mode decomposition (ACMD) [10] and Hilbert-Huang Transform (HHT) [11]. HHT combines empirical mode decomposition (EMD) and Hilbert spectral analysis. The calculation process of HHT is to decompose a signal into a set of intrinsic mode functions (IMFs) using EMD, and then extract instantaneous amplitude and frequency information by applying Hilbert transform (HT) to the IMF components. The frequency resolution of IMFs has adaptive filtering characteristics, and HT is an important demodulation tool. These will contribute to noise cleaning and stationarisation. The Hilbert envelope spectra obtained from IMFs can be also compared with the fault characteristic frequency to detect the presence of defects preliminarily. However, due to the endpoint effects and modal aliasing in EMD, Wu and Huang [12] proposed adding white noise to the original signal and calculating the means of IMFs repeatedly. The improved method is called ensemble empirical mode decomposition (EEMD). To extract weak defect features for the identification of bearing defects, Kumar et al. [13] established a non-parametric complementary ensemble empirical mode decomposition (NPCEEMD) based methodology.

After signal preprocessing, statistical features can be extracted as physical health indexes. These features have a certain correlation with the load, speed, and degradation degree of bearings, but each feature is only sensitive to a specific defect at a specific stage. Therefore, to comprehensively reflect the running state of the system, a health index that integrates multiple features has been proposed [14]. In the actual work process, even if the installation, lubrication, and maintenance all meet industrial standards, the performance of bearings deteriorates as fatigue accumulates. Data-driven fault diagnosis methods remove the requirement of prior knowledge and accurate dynamic models, and identify fault types by feature engineering and pattern recognition technology without dismantling mechanical devices. Korkmaz et al. [15] investigates the application of machine learning algorithms in prediction and classification of tool wear and its state. Vashishtha et al. [16] developed a robust intelligent fault diagnosis scheme of worm gearbox based on adaptive Convolutional Neural Networks (CNN). However, many diagnostic methods have weak interpretability. A Hidden Markov Model (HMM) reflects the correlation between hidden and observed states through probability graphs, its rich mathematical structure enables it to be reasonably explained and lays a good theoretical foundation for its application in the fields of health assessment and fault diagnosis. Ocak and Loparo [17, 18] explored online monitoring and fault diagnosis of rolling element bearings based on HMMs: An HMM was trained with normal state data to monitor equipment fault state based on the likelihood of online monitoring data in the model; HMMs were trained separately for normal and fault states, and the most matching state type was identified by comparing the likelihood of test data in each model. Jiang et al. [19] proposed adjacent singular value ratio features, and discussed a performance degradation evaluation method based on the combination of singular value decomposition and HMMs. In recent years, there has been a gradual increase in research on the improvement of HMM itself, which has improved the application ability of the model. Huang et al. [20] used predictive neural networks and intuitionistic fuzzy sets to optimize the selection of the initial value of the emission probability matrix, improving the accuracy of HMM-based fault diagnosis. To solve the problem of significant deviation between HMMs and actual system health diagnosis, Liu et al. [21] developed an improved degenerated HMM with a core of the quasi-power relation. Gámiz et al. [22] used HMMs to describe the evolution-in-time of a system and to estimate its characteristics when direct observations of the system state are not available. The above work optimized the parameter solving algorithm and application mode of the model, but did not overcome the structural limitations of the model. In fact, the Markov assumption, homogeneity assumption, and output independence assumption of HMMs result in weak generalization and unstable output.

The Mahalanobis Distance (MD) does not rely on the distribution of data and can eliminate the influence of dimensions [23], and its statistical characteristics have a certain degree of complementarity to probability measurement. There are high credibility and robustness requirements for the health assessment results of bearings, while the Dempster-Shafer theory of evidence can provide stable fusion judgment and accurate comprehensive measurement for uncertainty problems [24]. Therefore, an intelligent health assessment method that fuses HMMs and MD by the Dempster-Shafer theory of evidence is proposed in this paper. There are high generalization and accuracy requirements for the fault diagnosis results of bearings, while the Stacking algorithm can integrate multiple types of weak learners into a strong learner which can improve the generalization performance of the model [25]. Therefore, an intelligent fault diagnosis method that integrates HMMs by the Stacking algorithm is proposed in this paper. The layout of the rest of the paper is organized as follows: Section 2: Feature engineering presents signal processing and feature extraction techniques. Section 3: Models and methods introduces basic models and methods. Intelligent health assessment and fault diagnosis frameworks based on HMMs are presented in Section 4: Flow of health assessment and fault diagnosis. The XJTU-SY rolling element bearing accelerated life test datasets are used to verify the effectiveness of the health assessment and fault diagnosis methods in Section 5: Case studies. The conclusion of this paper is given in Section 6: Conclusion.

## 2. Feature engineering

Feature engineering and machine learning are two important aspects of data-driven methods. Feature engineering utilizes relevant knowledge in the field of data to construct feature spaces and prepare input samples for machine learning algorithms. The main processes of feature engineering for mechanical vibration signals include signal preprocessing, feature extraction, and dimensionality reduction.

### 2.1. EEMD

EMD reduces the complexity of signal processing by decomposing a signal into a series of stationary signals. EEMD can eliminate the mode mixing problem in all cases automatically, thus representing a major improvement of EMD. The main steps are as follows:

***Step 1*.** Add white noise sequences multiple times to the original signal *x*(*t*) to obtain a series of new signals

$$x_{m}(t)=x(t)+n_{m}(t),m=1,2,\ldots ,h,$$ (1)

where $n_{m}(t),m=1,2,\ldots ,h$ represents a series of Gaussian white noise, and *h* represents the number of times white noise has been added.

***Step 2*.** Exert EMD on each new signal *x*_*m*_(*t*) to obtain the decomposition results

$$x_{m}(t)=\sum_{i=1}^{n}c_{mi}(t)+r_{m}(t),m=1,2,\ldots ,h,$$ (2)

where *c*_*mi*_(*t*) is the *i*-th IMF of the *m*-th new signal, and *r*_*m*_(*t*) is the residual component of the *m*-th new signal.

***Step 3*.** Take the mean of the IMF and residual components of each new signal to obtain

$$c_{i}(t)=\frac{1}{h}\sum_{m=1}^{h}c_{mi}(t),i=1,\ldots ,n,$$ (3)

$$r(t)=\frac{1}{h}\sum_{m=1}^{h}r_{m}(t),$$ (4)

where *c*_*i*_(*t*) represents the *i*-th IMF obtained through EEMD, and *r*(*t*) represents the residual component.

### 2.2. Hilbert envelope spectrum

Hilbert envelope spectrum analysis is a resonance demodulation technique for stationary signals. Compared to the original non-stationary signal, the IMFs after decomposition are more suitable for Hilbert envelope spectrum analysis. The analysis process is as follows: Perform a 90° phase shift on *c*_*i*_(*t*), i.e

$$H[c_{i}(t)]=\frac{1}{\pi t}c_{i}(t)=\frac{1}{\pi }\int_{-\infty }^{+\infty }\frac{c_{i}(\tau )}{t-\tau }d\tau .$$ (5)

Take it and *c*_*i*_(*t*) as the imaginary and real parts of the analytic signal $c_{i}^{\prime }(t)$, respectively,

$$c_{i}^{\prime }(t)=c_{i}(t)+jH[c_{i}(t)].$$ (6)

The amplitude of the analytic signal is the envelope signal

$$A(t)=\sqrt{{c_{i}}^{2}(t)+H^{2}[c_{i}(t)]}.$$ (7)

The envelope spectrum can be obtained by frequency analysis of the envelope signal using the Fourier transform (FT).

### 2.3. Feature extraction and dimensionality reduction

The core of feature engineering is feature extraction and dimensionality reduction. Multi-domain features reflect the statistical characteristics of signals from different perspectives, which can more comprehensively reflect the state information of the system and better improve algorithm performance. The time-domain features mainly reflect the amplitude fluctuations, energy fluctuations, and waveform distribution of vibration signals. This paper intends to extract 10 time-domain features, including mean, standard deviation, root mean square (RMS), peak, skewness, kurtosis, crest factor, clearance factor, shape factor and pulse factor. The frequency domain features reflect the distribution of the main frequency band and the degree of dispersion and concentration of the spectrum. There are 6 frequency domain features to be extracted in this paper, including mean frequency, standard deviation frequency, root mean square frequency, frequency center, Skewness frequency, and Kurtosis frequency. Entropy features can better reflect the uncertainty and randomness of signal changes. There are 3 entropy features to be extracted in this paper, including approximate entropy, fuzzy entropy, and sample entropy. Meanwhile, to improve computational efficiency, the Fisher scoring algorithm [26] is used to reduce the dimensionality of the feature vectors by shortening the intra-class distance and increasing the inter-class distance of the reduced feature vectors.

Given the above theoretical analysis, the specific method for feature extraction and dimensionality reduction of vibration signals is to sample continuous signals at a fixed frequency to obtain a series of discrete data points and segment the signals into non-overlapping windows, i.e. equal-sized epochs, each containing the same number data points; Obtain the IMF components of each signal segment through EEMD, and then use Hilbert transform and fast Fourier transform (FFT) to obtain the envelope spectra; Extract time-domain and entropy features from the IMFs, and extract frequency-domain features from the envelope spectra; Use the Fisher scoring algorithm to sort the importance of the features and select the most informative features. The samples are represented as feature vectors after dimensionality reduction and can be used as input samples of the model, each corresponding to the current system running state. In the following, *n* denotes the number of samples and *p* denotes the feature dimension.

## 3. Models and methods

This section focuses on the basic concepts and application methods of HMMs. At the same time, to establish a fusion model, a brief introduction is given to DS evidence theory and MD. To establish an ensemble learning model, a brief introduction is given to Stacking and deep learning algorithm Long Short Term Memory (LSTM) network.

### 3.1. Hidden Markov Model

HMMs can be defined by the following parameters: number of hidden states *N*, the number of observable states *M* corresponding to each hidden state, initial probability distribution *π*, state transfer probability matrix *A* and emission probability matrix *B* or probability density function

$$b_{j}(O)=\sum_{m=1}^{M_{j}}c_{jm}N(O,u_{jm},U_{jm}),1\leq j\leq N,$$ (8)

where *O* is the sequence of observations, *N*(∙,∙,∙) is the Gaussian distribution density function, *M*_*j*_ is the number of Gaussian elements in the *j*-th state, *c*_*jm*_ is the mixing coefficient of the *m*-th Gaussian element in the *j*-th state, *u*_*jm*_ and *U*_*jm*_ is the mean vector and covariance matrix of the *m*-th Gaussian element in the *j*-th state, respectively.

HMMs mainly solves three problems in application, namely evaluation problem, decoding problem, and learning problem [20]. The evaluation problem refers to how to calculate the log-likelihood (LL) when parameters and observation sequences are known. The decoding problem refers to how to find the most likely state sequence when the observation sequence is known. Learning problem refers to how to adjust parameters to maximize the emission probability of the observation sequence. The classic methods to solve these three problems are the forward-backward algorithm, the Viterbi algorithm, and the Baum-Welch algorithm [21].

### 3.2. The Dempster-Shafer theory of evidence

In this section, the main concepts of the Dempster-Shafer theory of evidence are briefly recalled. We refer to a set *U* that contains finite and incompatible propositional elements as the frame of discernment. Define the basic probability distribution function *m* from the power set of *U* to the interval [0,1] that satisfies

$$\left\{ \begin{array}{l} m(\emptyset )=0,\\ \sum_{E\in 2^{U}}m(E)=1, \end{array}\right.$$ (9)

where *m*(*E*) represents the degree of support for proposition *E* and is called the basic credibility. If *m*(*E*)>0, then *E* is called the focal element. Assume *m*_1_ and *m*_2_ are independent bodies of evidence under the same recognition framework, and define combination basic probability distribution functions *m* = *m*_1_⊕*m*_2_. The combination rule is

$$m(G)=\{\begin{array}{l} \sum_{\begin{array}{l} E,F\in 2^{U}\\ E\cap F=C \end{array}}\frac{m_{1}(E)\cdot m_{2}(F)}{1-L},\;C\neq \emptyset \\ 0,\;C=\emptyset \end{array}$$ (10)

where $L=\sum_{\begin{array}{l} E,F\in 2^{U}\\ E\cap F=\emptyset \end{array}}m_{1}(E)\cdot m_{2}(F)$ is the normalization factor. If *L* = 1, it is considered that *m*_1_ and *m*_2_ are completely contradictory and cannot be combined.

### 3.3. Mahalanobis distance

The calculation of MD mainly includes the inverse matrix method, adjoint matrix method, and Gram-Schmidt orthogonalization method. The covariance matrix of high-dimensional features often tends to be singular, while the adjoint matrix requires a large amount of computation. Therefore, this paper considers using the Gram-Schmidt orthogonalization method to calculate MD.

Let the column vectors of the feature matrix after standardization and Gram-Schmidt orthogonalization are $U_{j}={\left(u_{1j},u_{2j},\ldots ,u_{nj}\right)}^{T},j=1,2,\ldots p$, then the calculation formula for MD is

$${\mathrm{MD}}_{i}=\frac{1}{p}(\frac{u_{i1}^{2}}{s_{1}^{2}}+\frac{u_{i2}^{2}}{s_{2}^{2}}+\ldots +\frac{u_{ip}^{2}}{s_{p}^{2}}),i=1,2,\ldots ,n,$$ (11)

where *s*_1_,*s*_2_,…,*s*_*p*_ represents the standard deviation of *U*_1_,*U*_2_,…,*U*_*p*_.

### 3.4. Stacking

Stacking is an ensemble learning algorithm that integrates weak learners into strong learners to improve generalization performance and training effectiveness. The composition of Stacking includes base learners and meta-learners. Base learners perform preliminary learning, prediction, and testing on initial samples, while the meta learner performs secondary training and testing based on the output of the base learners. Although Stacking has a cumbersome trial-and-error process, it is a highly effective approach for increasing the predictive performance of machine learning. The specific steps of the Stacking ensemble algorithm are: Divide the initial training and test set; Divide the initial training set into *K* parts, select each part as the prediction set in sequence, and treat the remaining *K*-1 parts as the training subsets simultaneously; For *K* training subsets, train the base learners sequentially to obtain the test results of the corresponding prediction set and all test sets; Combine the test results of *K* prediction sets as the training set of the meta learner, and then take the mean of the test results of *K* test sets as the test set of the meta learner; Train the meta learner and test its classification performance.

### 3.5. LSTM network

To extract the deep-level features of model output, the deep learning algorithm LSTM network is selected as the meta-learner. LSTM network is an improvement of Recurrent Neural Network (RNN), which has good analytical ability for time series data. Its gating structure consists of an input gate, forget gate, and output gate, which controls information selection, input update, and output. This enables LSTM to have a long-term memory function and effectively solve the problems of gradient explosion and vanishing during RNN training, greatly enhancing the accuracy of RNN.

## 4. Flow of health assessment and fault diagnosis

Combining artificial intelligence methods, HMMs are applied in the bearing health assessment and fault diagnosis. The procedures of these two applications are introduced, and the development and implementation of two prognostic frameworks are discussed in the following sections.

### 4.1. Health assessment framework

Health assessment plays a fundamental role in the implementation of PHM and is a prerequisite for CBM. The likelihood of an HMM is effective in tracking the bearing condition. Firstly, select training samples from the initial stage and train an HMM. Secondly, consider 10 consecutive samples as one observation sequence and extract life cycle observation sequences. Finally, input them into the trained model to obtain the corresponding LL sequence. The larger the LL, the higher the probability that the current state is normal. The smaller the LL, the lower the probability that the current state is normal. An HMM uses sliding observation sequences composed of past and current samples as input which reflects the correlation and continuity of state changes, so the performance evaluation results are smoother. An HMM has high requirements for data distribution because of its Markov assumption, homogeneity assumption, and output independence assumption, while MD does not rely on the data distribution and can eliminate the influence of dimensionality. MD reflects the similarity between the current sample to the training sample space, and its statistical characteristics form a good complementarity to probability measures. Therefore, the fusion of the two models has stronger generalization and robustness.

To make the evaluation results universal, an evaluation function *f*:*R*→[0,1] is used to map the performance degradation evaluation results to the internal [0,1]. The final results are called health indexes (HIs). The closer HI is to 1, the more stable the system is running, while the closer HI is to 0, the more unstable the system is running, and there is even a risk of shutdown. Using the empirical evaluation function y = arctan*x*, the formula for calculating HI based on HMM is

$${\mathrm{HI}}_{1}=1+2\times \arctan(\alpha \times (\mathrm{LL}-\beta ))/\pi ,$$

and the formula for calculating HI based on MD is

$${\mathrm{HI}}_{2}=1-2\times \arctan(\gamma \times \mathrm{MD})/\pi ,$$

where *α*,*β*,*γ* are adjustment factors.

If HIs obtained from the two models are relatively close, it indicates that the obtained results have high credibility. If there are certain differences between two HIs, inference rules should be adopted to obtain more credible comprehensive results. The DS theory can effectively fuse multiple decisions and accurately measure uncertainty problems. As an application, the DS theory is used to fuse HIs.

The stability of the HI sequence of the training samples reflects the predictive ability of the evaluation methods. Therefore, we calculate the variance of the HI sequence obtained by the two methods after maximum and minimum normalization and allocate global confidences *γ*_*i*_,*i* = 1,2 according to the size of the variance. We can obtain $m_{i}(S_{1},S_{2},\Theta ,\emptyset )=(\gamma_{i}\cdot {\mathrm{HI}}_{i},\gamma_{i}\cdot (1-{\mathrm{HI}}_{i}),1-\gamma_{i},0),$ *i* = 1,2, where *S*_1_ and *S*_2_ represent normal and abnormal states, respectively. By Formula (10), HI = *m*(*S*_1_) can be calculated. To divide the normal and abnormal samples, a control chart is used to determine the normal sample interval. Assume the mean of HIs of the training samples is *μ* and the standard deviation is *σ*. if HI falls within the interval [*μ*−3*σ*,*μ*+3*σ*], it indicates that the current sample is normal, otherwise, it is an abnormal sample. Fault occurrence point and the degree of performance degradation can be also determined subsequently.

The health assessment framework proposed in this paper only requires a portion of normal samples as training samples and does not require much prior knowledge and massive historical operating data. HIs can be used to locate the fault occurrence point and quantify the degradation state timely and accurate. Moreover, it can help to accumulate a fault mode library for fault diagnosis. The steps of a real-time health assessment framework for rolling element bearings based on HMMs are as follows:

***Step 1*.** Sample the original signal at a fixed frequency and divide it into signal epochs. Decompose each signal by EEMD to obtain a series of IMFs, and extract the Hilbert envelope spectrum on each component. Extract multi-domain features from preprocessed signal epochs and reduce their dimensionality by the Fisher scoring algorithm. The feature vectors are used as input samples for the model.

***Step 2*.** Select training samples, train an HMM, and then calculate the LL sequence of the life cycle samples; Calculate the mean and standard deviation of the training samples, standardize the life cycle samples according to this mean and standard deviation, and calculate the corresponding MD sequence after Gram-Schmidt orthogonalization.

***Step 3*.** Based on the LL sequence and MD sequence, calculate the HI sequence under two different models, and use DS evidence theory to obtain the life cycle fusion results. Calculate the mean and standard deviation of the fusion HI of the training samples, and use a control chart to determine the normal sample interval. Determine fault occurrence points and distinguish the normal and abnormal samples.

### 4.2. Fault diagnosis framework

In the health assessment framework, bearing conditions can be continuously monitored and early warning of impending faults can be provided. As the degree of degradation intensifies, timely fault diagnosis should be carried out to develop scientific and effective maintenance decision-making and gradually establish a fault mode library. During the data accumulation phase, normal and abnormal samples should be distinguished, and all types of typical faults should be determined through disassembly inspection. After the mode library is established, fault diagnosis can be carried out through signal analysis and pattern recognition techniques without dismantling the machine. Diagnostic problems are essentially classification problems, and fusion methods can integrate the classification results of multiple models to improve the stability of classification models. However, the overall accuracy is often at the average level. In recent years, the application research of ensemble learning in fault diagnosis models has gradually deepened. Ensemble learning can achieve complementary advantages of different models, further improving classification accuracy and robust performance. An HMM has outstanding classification ability, but the selection of hyper parameters is also relatively difficult. The method of multiple partitioning of the training set and retraining the output results through Stacking can discover the optimal performance of the base learner, correct misclassification, and reduce the misjudgment probability. Therefore, this section attempts to establish a fault diagnosis framework using the ensemble HMM.

The process of using HMMs for fault diagnosis alone is: divide the data into a training set and a test set; Train HMM classifiers for various types of fault; Input each test sample into each classifier to obtain a column vector of LL. The category corresponding to the maximum value in the column vector is the diagnostic result. The Stacking algorithm provides a novel training strategy: the training set is divided into *K* classes with one class selected as the prediction set and the other *K*-1 class as the new training set; The new training set is used to train HMMs, and the prediction and test sets are tested; Continuously select new training sets, repeat this process *K* times, and take the mean of the test results for *K* test sets; Select different hyper parameters and repeat the above process *M* times, so that the test result of each prediction set is a vector sequence, and the test result of the test set is also a vector column, which is used as the training set and test set for the meta learner. To extract the deep-level features of HMM outputs, the deep learning method LSTM network is selected as the meta-learner. The main steps of an automated fault diagnosis framework for rolling element bearings based on HMMs are:

***Step 1*.** Sample the original signal at a fixed frequency and divide it into signal epochs. Decompose each signal by EEMD to obtain a series of IMFs, and extract the Hilbert envelope spectrum on each component. Extract multi-domain features from preprocessed signals and reduce their dimensionality through the Fisher scoring algorithm. The feature vectors on each signal epoch are used as input samples for the model.

***Step 2*.** Divide the initial training set and test set, obtain the HMM-based prediction set and test set output results using the Stacking-based learner training method, and combine them into the training and test sets of the meta learner.

***Step 3*.** Train the LSTM network with the meta-learner training sets obtained in Step 2, and use the trained ensemble model to test the meta-learner test sets.

To quantitatively evaluate the classification performance of the fault diagnosis model, the following evaluation indicators are adopted.

$$\mathrm{Accuracy}=\frac{\mathrm{TP}+\mathrm{TN}}{\mathrm{TP}+\mathrm{FP}+\mathrm{TN}+\mathrm{FN}},$$

$$\mathrm{Precision}=\frac{\mathrm{TP}}{\mathrm{TP}+\mathrm{FP}},$$

$$\mathrm{Recall}=\frac{\mathrm{TP}}{\mathrm{TP}+\mathrm{FN}},$$

$$F1=\frac{2\times \mathrm{Precision}\times \mathrm{Recall}}{\mathrm{Precision}+\mathrm{Recall}},$$

where TP is the number of normal samples correctly detected as normal (True Positives), FP is the number of abnormal samples that are detected as normal (False Positives), FN is the number of abnormal samples that are detected as abnormal (False Negatives), TN is the number of normal samples that are detected as abnormal (True Negatives).

**Fig 1:**
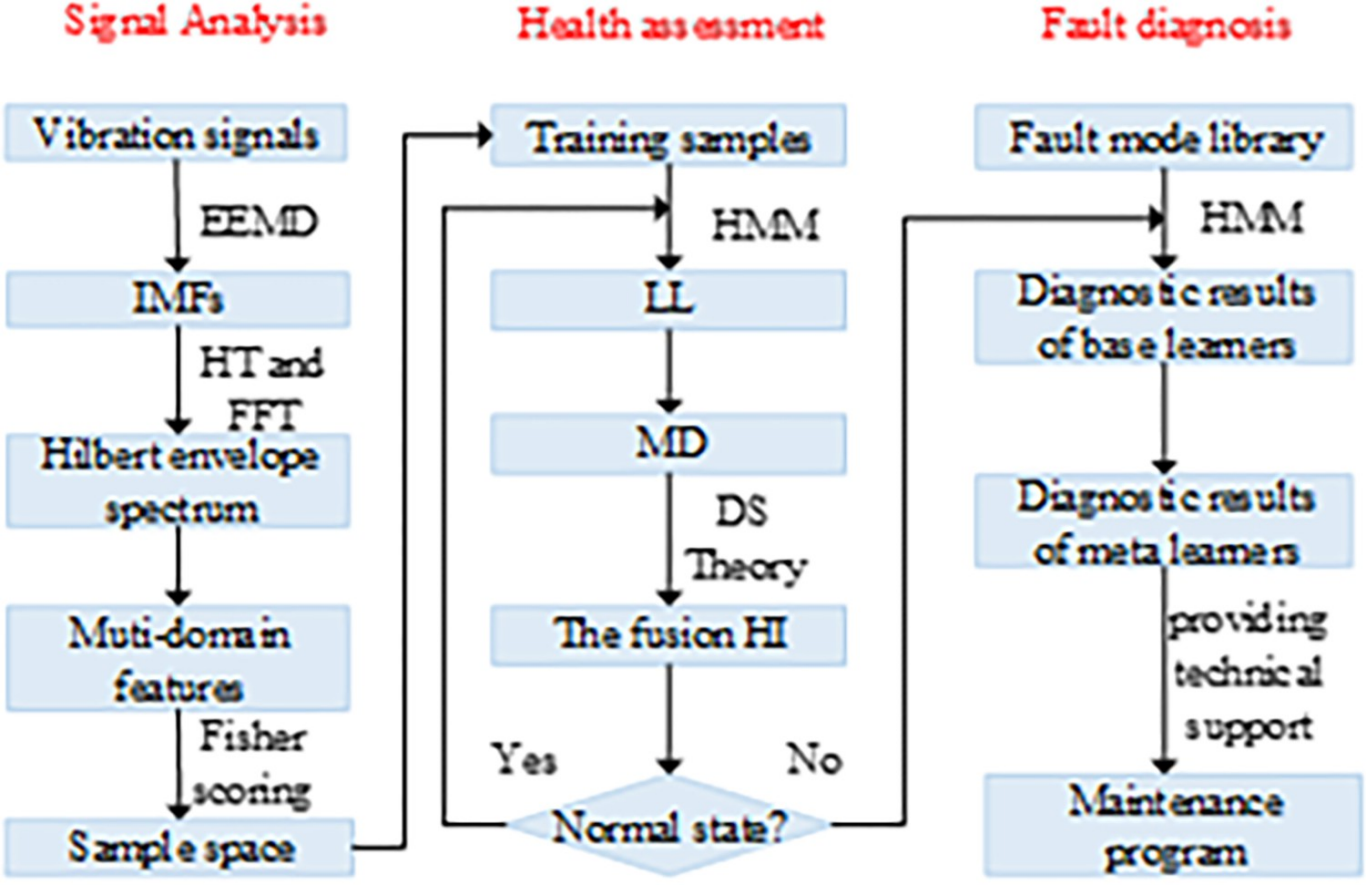
Health assessment and fault diagnosis process.

Health assessment and fault diagnosis are inseparable and interrelated processes. During the operational phase of the system, continuous health assessments should be conducted, and timely fault diagnosis should also be carried out when state degradation is detected. The complete flow of health assessment and fault diagnosis is shown in Fig 1.

## 5. Case studies

The proposed bearing health assessment and fault diagnosis methods are validated using XJTU-SY bearing datasets provided by the Institute of Design Science and Basic Component at Xi’an Jiaotong University (XJTU) and the Changxing Sumyoung Technology Co., Ltd. (SY).

### 5.1. A brief introduction to the datasets

**Fig 2:**
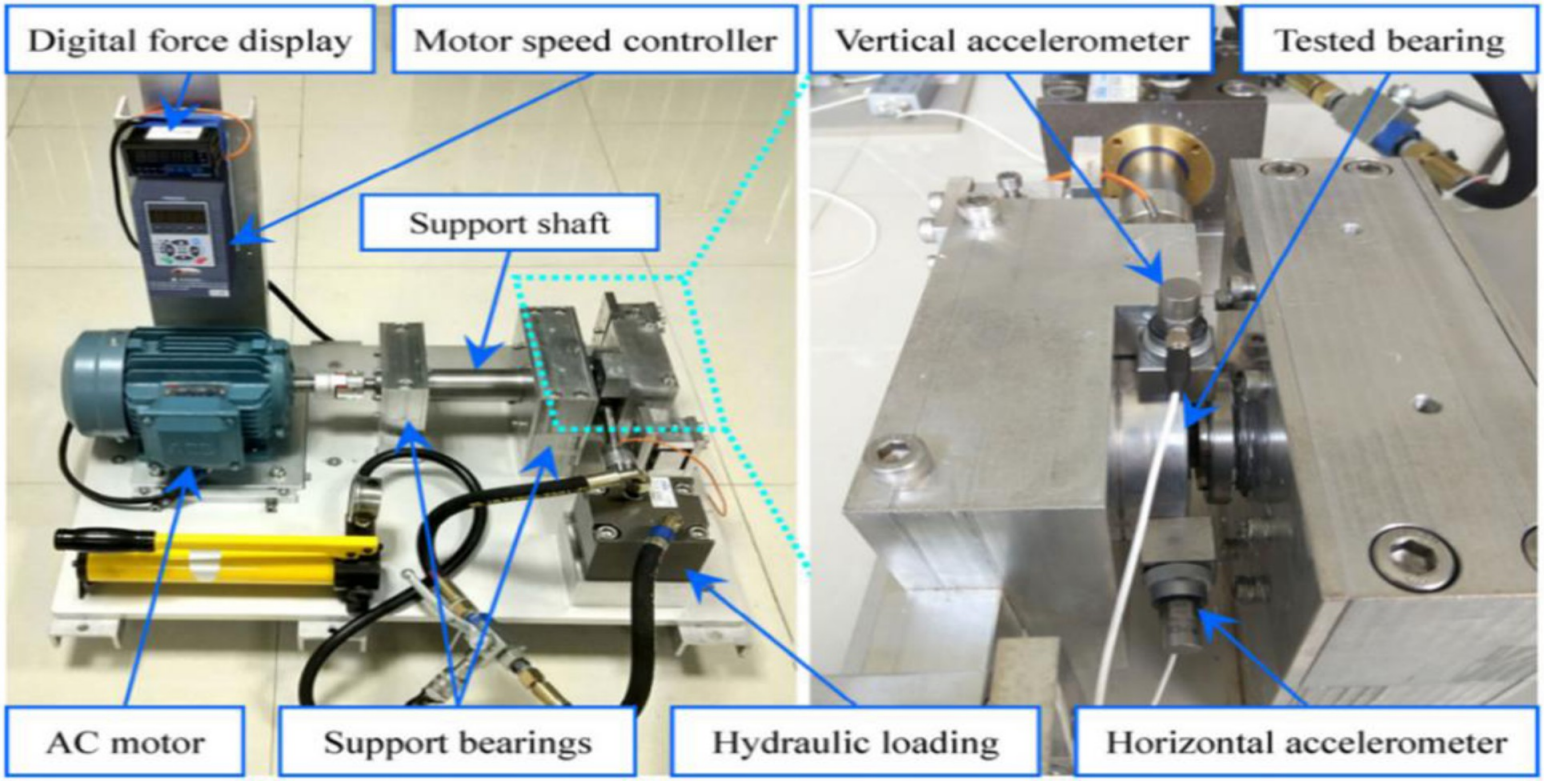
Testbed of rolling element bearings.

The bearing testbed for collecting complete run-to-failure data of 15 rolling element bearings is shown in Fig 2. This testbed is composed of an alternating current (AC) induction motor, a motor speed controller, a support shaft, two support bearings (heavy-duty roller bearings), a hydraulic loading system, and so on. This testbed is designed to conduct accelerated degradation tests of rolling element bearings under different operating conditions (i.e., different radial forces and rotating speeds). The radial force is generated by the hydraulic loading system and the rotating speed is set and kept by the speed controller of the AC induction motor. The type of tested bearings is LDK UER204, and the detailed parameters are given in Table 1. A total of 3 different operating conditions were set in the accelerated degradation experiments, and 5 bearings were tested under each operating condition. The details of operating conditions for bearings are shown in Table 2. The sampling period is equal to 1 min and each sampling lasts for 1.28 seconds. The sampling frequency is set to 25.6 kHz, so 32768 data points in 1.28s are recorded.

**Table 1:** Bearing parameters.

Number of rolling balls	Contact angle	Rolling body diameter	Bearing diameter	Inner race raceway diameter	Outer ring raceway diameter
8	0 degree	7.92mm	34.55mm	29.30mm	39.80mm

**Table 2:** The operating conditions for bearings.

Condition number	1	2	3
Speed (r/min)	2100	2250	2400
Radial force (kN)	12	11	10

Inner and outer ring faults are the main failure modes. This paper mainly selects specific datasets for analysis. For the name of bearing i_j in Table 3, i denotes the index of the working condition and j denotes the case index. The fault characteristic frequencies of two fault types under three operating conditions can be calculated from the parameter values in Table 1. The calculation results are shown in Table 4.

**Table 3:** Experimental datasets.

Fault Types	Data sources
Inner ring fault	Bearing2_1, Bearing3_3, Bearing3_4
Outer ring fault	Bearing1_1, Bearing1_2, Bearing2_2, Bearing3_1

**Table 4:** Fault characteristic frequency.

Condition number	1	2	3
Inner ring fault characteristic frequency (Hz)	172.09	184.38	196.68
Outer ring fault characteristic frequency (Hz)	107.91	115.62	123.32

### 5.2. Health assessment results and discussion

**Fig 3:**
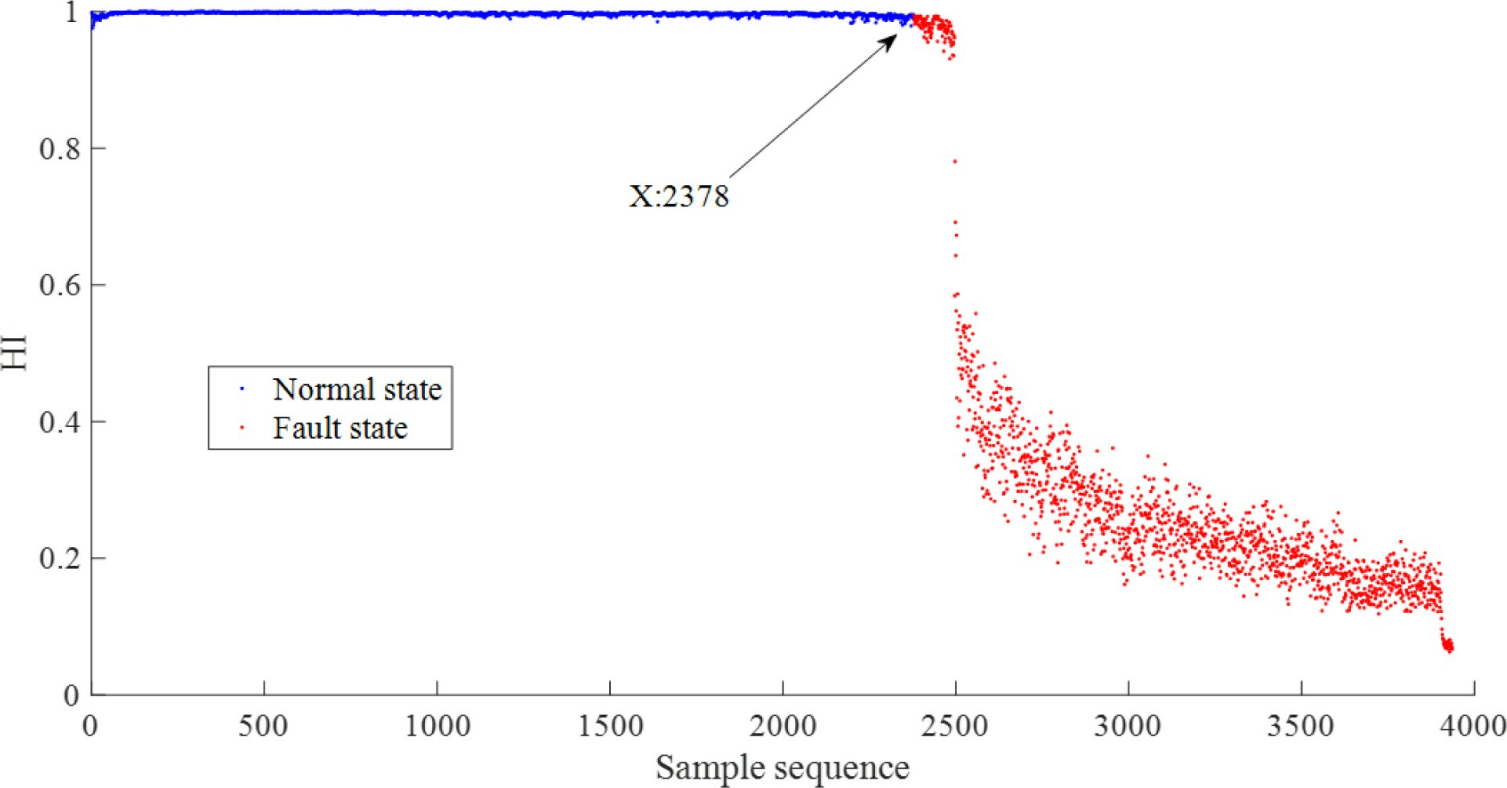
Health assessment results of Bearing1_1.

The vibration signals in the XJTU-SY bearing datasets are sequentially divided into non-overlapping epochs. Each epoch contains 1024 data points and is decomposed by EEMD method. By sorting the correlation coefficients with the original signals, the four most sensitive IMFs of the epochs and their Hilbert envelope spectra are obtained. Based on the features listed in Section 2.3: Feature extraction and dimensionality reduction, 76 features are extracted from each epoch. Ultimately, 3936×76 feature vectors obtained from Bearing1_1 form a sample set. The first 1000 samples are selected as the training samples, assuming that the system runs smoothly and normally before the moment corresponding to the 1000th sample. Assuming that each observation sequence contains 10 samples, an HMM is trained with 1000 training samples to obtain the life cycle assessment results. The model has three states and two Gaussian distributions are used in the output map for each state. The mean and standard deviation of the training samples are calculated as 0.999 and 0.499. Adjustment factors *α*,*β*,*γ* are adaptively estimated as 0.002, 331.883, and 0.020. Therefore, the HIs based on HMM and MD can be obtained. Subsequently, global confidences *γ*_*i*_,*i* = 1,2 are assigned as 0.995 and 0.980. Above all, the fusion HI can be obtained by using DS theory. The mean and the standard deviation of the fusion HI of the first 1000 samples are calculated as 0.980 and 0.002 respectively, then control charts can be determined to divide normal and faulty samples, the life cycle assessment results of bearing1_1 are shown in Fig 3.

**Fig 4:**
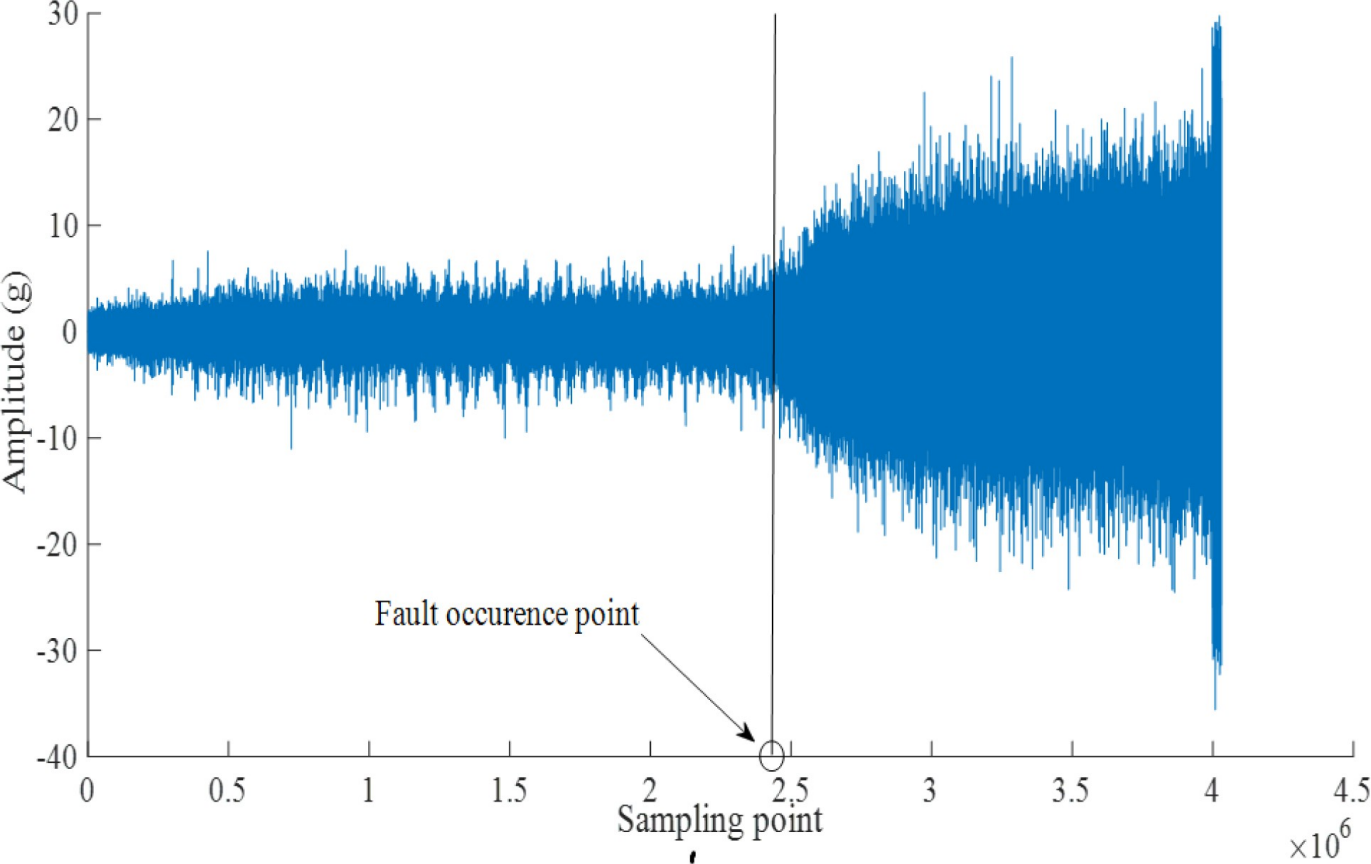
Vibration signal of Bearing1_1.

In Fig 3, The blue point indicates that the current state is normal, while the red point indicates that the current state is faulty. Two states are segmented by the 2378th sample. The corresponding fault occurrence point in the original signal is shown in Fig 4. HI remains relatively stable before the 2378th sample, and then sharply decreases to below 0.6 with intense fluctuations. This situation indicates that it is undergoing a severe stage of wear and tear. Eventually, HI remains at an extremely low level and does not rebound, indicating that it has almost failed.

**Fig 5:**
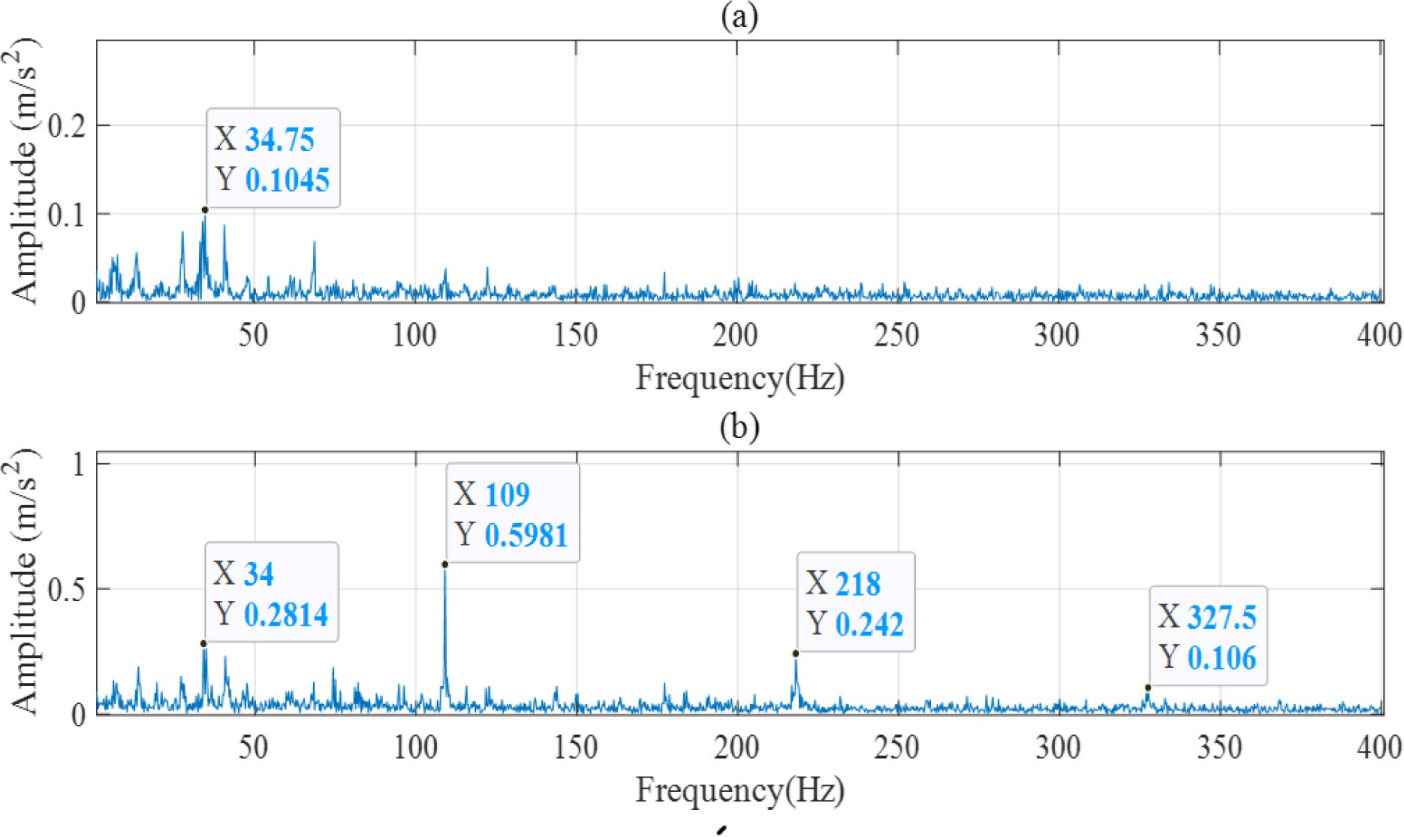
Hilbert envelope spectrum.

As shown in Fig 4, the signal amplitude is relatively stable and low before the fault occurred point, but oscillates after the fault occurrence point. In the final stage, the amplitude even exceeds 20g. These can be reflected more clearly and intuitively through HI. To obtain information about fault style, the Hilbert envelope spectra of the signals after EEMD are plotted in Fig 5. Fig 5(A) shows the Hilbert envelope spectrum before the fault occurrence point, and Fig 5(B) shows the spectrum after the fault occurrence point.

**Fig 6:**
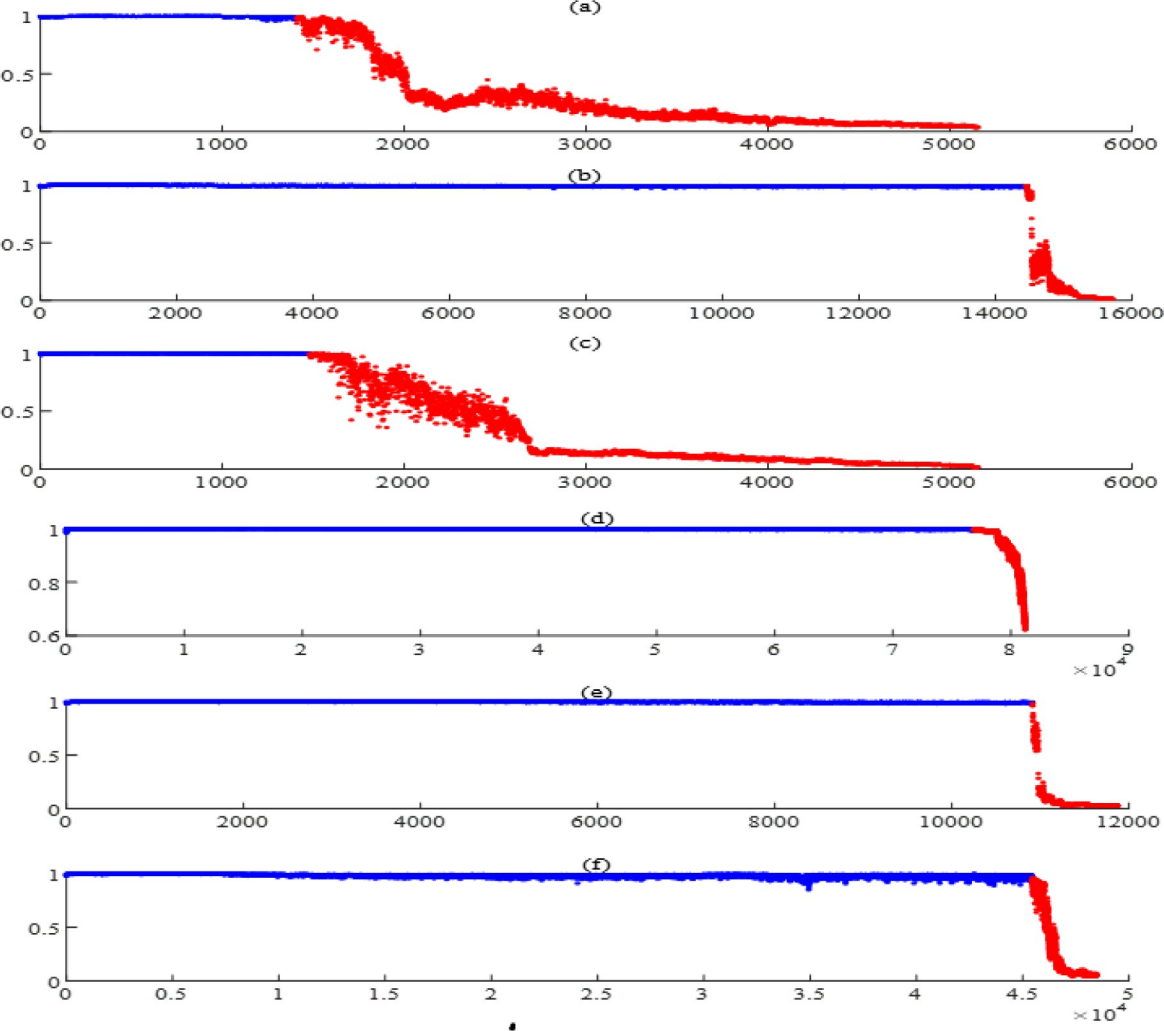
Health assessment results. (a) Bearing1_2, (b) Bearing2_1, (c) Bearing2_2, (d) Bearing3_1, (e) Bearing3_3, (f) Bearing3_4.

Except for the rotating frequency of 34Hz, there is no obvious fault frequency before the fault occurrence point, and the fault frequency of the outer ring and its frequency doubling are very obvious after the fault occurrence point, indicating that health assessment can effectively distinguish between normal and faulty samples and has good predictive ability. To reduce computational complexity, the Fisher scoring algorithm is used to reduce the feature dimension and obtain evaluation results for other bearings, as shown in Fig 6. It can be observed that each bearing has undergone a process from stable operation to gradual degradation, which conforms to the general law of bearing fatigue failure.

### 5.3. Fault diagnosis results and discussion

To demonstrate the effectiveness of the proposed fault diagnosis model, a classification experiment is also conducted on XJTU-SY bearing datasets. In this experiment, three bearing states are analyzed, which include normal condition (NC), inner race fault (IRF), and outer race fault (ORF). The sample acquisition method is the same as in Section 5.2: Health assessment results and discussion. The initial training set and test set are taken from different bearings. The specific method is to select one dataset from the inner race fault datasets and one dataset from the outer race fault datasets to form the training set, and separate the normal and fault samples of both; The remaining datasets serve as the test set, and also separate the normal and fault samples; 200×3 = 600 model inputs are randomly selected from the training set for three bearing states, and 200× 3 = 600 model inputs are also randomly selected from the test set. The selection of the training and test sets is also random, and one of the selection methods is shown in Table 5.

**Table 5:** The training set and test set.

Datasets	Bearing states			
	NC		IRF	ORF
Training set	Bearing2_1	Bearing1_1	Bearing2_1	Bearing1_1
Test set	Bearing3_3	Bearing1_2	Bearing3_3	Bearing1_2
	Bearing3_4	Bearing2_2	Bearing3_4	Bearing2_2
		Bearing3_1		Bearing3_1

**Fig 7:**
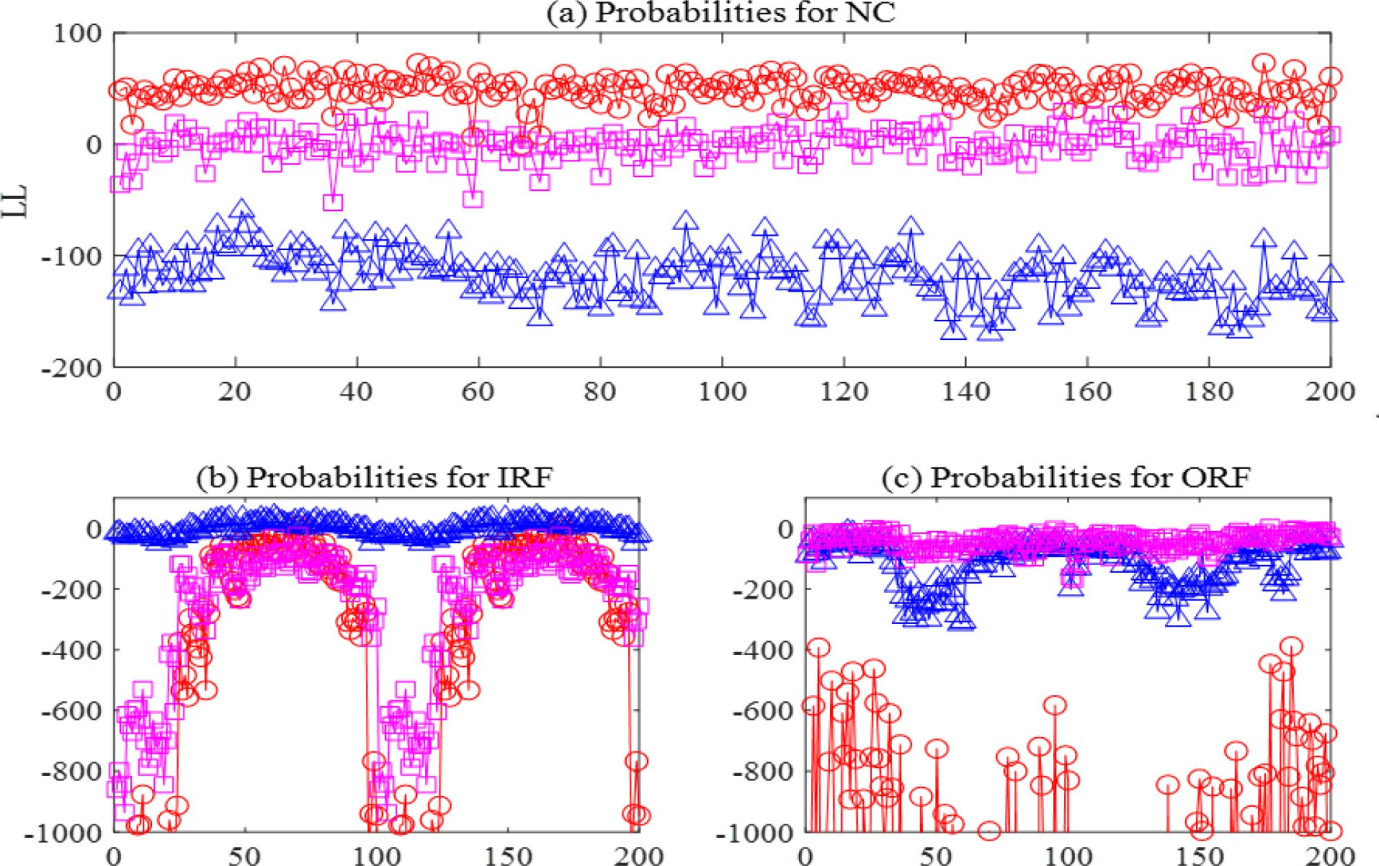
Base learners for fault diagnosis.

**Fig 8:**
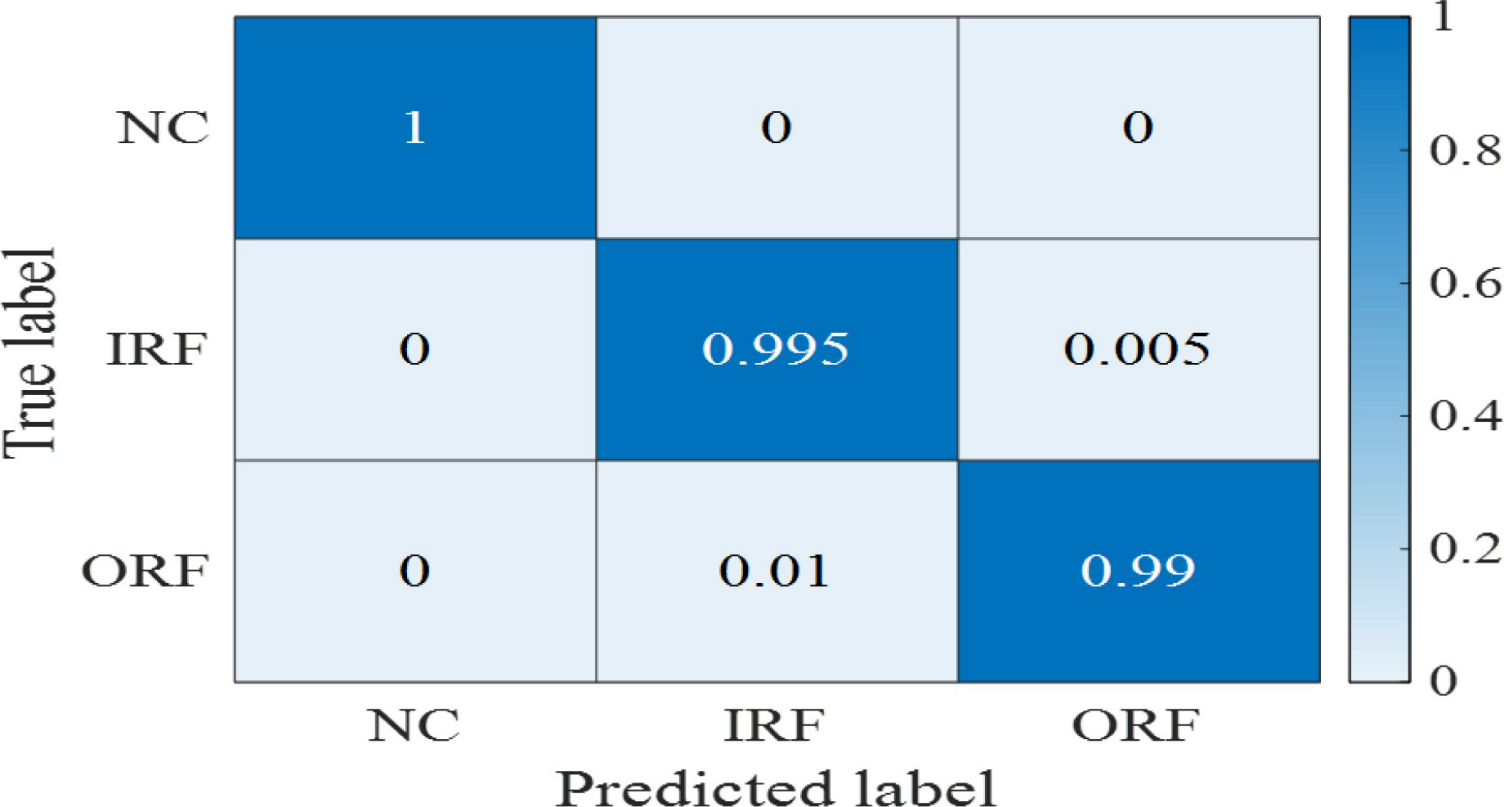
Confusion matrix of fault diagnosis using ensemble learners.

To solve the difficulty of selecting hyper parameters, the number of states for HMMs is set to 3, 4 and 5, and the number of Gaussian distributions for each state is set to 2 and 3. The initial training set will be divided into 5 training subsets, so the number of base learners is 3×2×5 = 30. After training and testing with the base learner, and then training and testing with the meta learner LSTM, the final diagnostic result can be obtained. Fig 7(A) shows the probabilities of the normal conditions, inner race fault, and outer race fault given the model for the normal condition. The probabilities of the normal conditions are separable from the probabilities of the faulty conditions. Fig 7(B) and Fig 7(C) show the probabilities given the model for inner race and outer race fault conditions, respectively. To visually display the classification results of meta-learners, a confusion matrix is created based on the test results, as shown in Fig 8.

In addition, 50 repeated experiments are conducted. Compared with the traditional HMM (base Learners), the specific results of evaluation indicators for testing in the ensemble HMM (meta Learners) are shown in Table 6. After secondary training, the average accuracy rate increases by 1.66% from 97.95% to 99.61%. Other evaluation indicators are also improved to a certain extent, and the test results are more stable. Meanwhile, health states are always fully recognized, and only a few two faulty states are misclassified from each other, so precision and recall are equal.

**Table 6:** The test results of two-stage learners.

Models	Accuracy	Precision	Recall	F1
Base Learners	0.9795±0.0062	0.9785±0.0055	0.9785±0.0061	0.9789±0.0059
Meta Learners	0.9961±0.0010	0.9951±0.0011	0.9951±0.0011	0.9951±0.0011

The computational complexity of ensemble HMM is a multiple of that of a single HMM due to repeated training, but it also brings a significant improvement in accuracy. The results of the experiment confirms that the method has certain anti-interference and generalization capabilities under variable operating conditions and with insufficient sample size. It has important guiding significance for establishing a fault mode library.

## 6. Conclusion

Health assessment and fault diagnosis are important means of system condition monitoring of rolling element bearings and are the technical bases for formulating maintenance plans. In this paper, intelligent health assessment and fault diagnosis frameworks for rolling element bearings based on HMMs are proposed and experiments on public datasets are performed to verify their effectiveness. Experimental results demonstrate that: By using EEMD and Hilbert envelope spectrum analysis, non-stationary signals can be decomposed into stationary signals effectively and fault characteristic frequency can be extracted accurately; The proposed HI can comprehensively reflect the degradation degree and the correlation of state changes. Normal and abnormal samples can be divided through control charts, so a fault model library can be gradually established; The ensemble HMM can integrate the advantages of learners with different hyper-parameters, reduce the misjudgment probability, and further improve classification accuracy. The method proposed in this paper has the following advantages for applications in manufacturing enterprises: Strong applicability for different operating conditions; Convenient and concise to use, avoiding excessive parameter tuning and reliance on prior knowledge; Beneficial for the accumulation of fault data. These findings provide theoretical support for the engineering application of rotating equipment intelligent operation and precise maintenance. In future research, bearing reliability analysis and maintenance decision-making will be selected as key directions.
